# Sternal Wound Complications: Results of Routine Use of Negative Pressure Wound Therapy

**DOI:** 10.21470/1678-9741-2019-0242

**Published:** 2020

**Authors:** Andrea De Martino, Federico Del Re, Giosuè Falcetta, Riccardo Morganti, Giacomo Ravenni, Uberto Bortolotti

**Affiliations:** 1 Section of Cardiac Surgery, University Hospital, Pisa, Italy.; 2 Section of Statistics, University Hospital, Pisa, Italy.

**Keywords:** Sternotom, Patient Discharge, Duration of Therapy, Sternum, Hospitalization, Infections, Postoperative Complications

## Abstract

**Introduction:**

Negative pressure wound therapy (NPWT) has significantly improved outcomes in individuals with superficial and deep sternal wound dehiscence (SWD). We report our experience with NPWT to evaluate factors influencing effectiveness, duration of treatment and postoperative hospital stay.

**Methods:**

We reviewed 92 patients with postoperative SWD following a median sternotomy. Patients were divided into 2 groups: those with a superficial SWD (Group 1; 72, 78%) and those with a deep SWD (Group 2; 20, 28%). Group 1 was further divided into 3 subgroups based on NPWT duration.

**Results:**

In both groups, none of the preoperative characteristics examined showed a significant association with longer NPWT duration. In Group 2, there was a trend for postoperative bleeding and neurological complications to be associated with longer treatment duration. In the entire series, staph infection resulted a weak predictor of NPWT duration. In each Group 1 subgroup and in Group 2, treatment days were compared with duration of hospitalization until discharge. Mean post-NPWT hospital stay was 6 days in subgroup 1, 12 days in subgroup 2 and 20 days in subgroup 3 (*P*<0.0001). At a median 3-year follow-up, there were 4 late deaths, none related to wound complications. No cases of SWD recurrence were observed.

**Conclusion:**

Our results confirm the effectiveness of NPWT in SWD management, while excessive treatment duration might have a negative impact on the length of hospital stay. Further studies are needed to define an optimal use of NPWT protocol.

**Table:** 

Abbreviations, acronyms & symbols	
BMI	= Body mass index
COPD	= Chronic obstructive pulmonary disease
CPB	= Cardiopulmonary bypass
NPWT	= Negative pressure wound therapy
SWD	= Sternal wound dehiscence
VAC	= Vacuum-assisted closure

## INTRODUCTION

Wound complications following median sternotomy are not so infrequent and, depending on their severity, may lead to devastating consequences that adversely affect patient outcomes^[1]^. Several treatments for sternal wound dehiscence (SWD) have been reported in the past, including debridement and immediate wound closure or irrigation and delayed closure^[2]^. In 1997 Argenta and Morykvas reported their clinical experience with negative pressure wound therapy (NPWT) as a new method for wound control and treatment^[3]^. Subsequently, this method has been applied to treat SWD with favorable results and recently it has been shown that NPWT reduces mortality and costs and should be considered as first-choice treatment and as a bridge to sternal closure in patients with sternal wound complications^[4]^. We have reviewed our experience with NPWT at our institution to evaluate factors influencing its effectiveness, treatment duration and interval between SWD and wound healing and patient discharge.

## METHODS

### Study Design

We have reviewed our institutional database of patients undergoing open-heart procedures through a median sternotomy with standard cardiopulmonary bypass (CPB) at our institution from January 2004 to December 2016. The aim of this study was to identify and analyze patients who developed postoperative wound infections and to evaluate the effectiveness of NPWT in their early and late outcomes. This study was approved by our local Ethical Committee without the need for patient consent owing to its retrospective nature.

### Definition of Terms

SWD was defined as 1) superficial, when only the cutaneous or subcutaneous layers were involved and 2) deep, when dehiscence reached the sternal surface with or without bone instability, fracture or separation of the sternal halves, with no evidence of mediastinitis. In the presence of negative or positive cultures of wound tissue, SWD was considered either sterile or infected. The diagnosis of mediastinitis was based according to the previously reported definition^[5]^.

### Preoperative Treatment

All patients were prepped and covered with disposable drapes. Antibiotic prophylaxis consisted of a single dose of intravenous cefazolin (2 g) within 60 minutes of the sternal incision and repeated at the end of CPB. In case of penicillin allergy, vancomycin was used^[6,7]^.

### Sternal Closure

After positioning of chest drains, hemostasis of the sternal halves was obtained with electrocautery and use of sterile bone wax. Sternal closure was accomplished by steel wires, adding since 2014 thermoreactive nitinol clips (Flexigrip; Praesidia SRL, Bologna, Italy) in case of fragile sternum, in obese patients and in those with a large chest. In the end, the wound was covered with plain sterile gauze.

### Wound Treatment

Following surgery, daily inspection of the wound was performed. Chest tubes were usually removed on postoperative day 2. In the ward, particular attention was given to the appearance of wound abnormalities. In such cases, the wound was partially reopened to drain any collection of subcutaneous material and culture swabs taken. Moreover, when particular bone fragility was reported in obese patients and those with a large chest, a Posthorax® Pro (Epple, Inc., Vienna, Austria) corset was applied and its use during the first month after discharge was strongly recommended.

### Wound Dehiscence

In the presence of a limited (<10 cm), superficial and sterile SWD, the wound was daily filled with iodoformic gauze and later closed surgically when deemed clean and dry. More extensive SWDs were treated taking the patient to the operating room, where wound debridement was carried out under sterile conditions, removing any foreign material and steel wires in case of sternal instability or fracture. Subsequently, NPWT was started using the KCI VAC® device (KCI Inc., San Antonio, TX, USA), which is made of a polyurethane foam dressing to apply uniform negative pressure over the field to eliminate any fluid collection. If there was space between sternal edges, right ventricle was protected with autologous tissue, paraffin gauze or porcine pericardium. The foam was tailored to fit the wound anatomy before its insertion. VAC was usually replaced every 24-72 hours, according to the features and amount of drainage, when a fragment of the wound was sent for culture. Antibiotic administration was started at the time of first culture and then modified based on microbiological results. NPWT suspension was based on the following criteria: 1) presence of 2 consecutive negative wound cultures; 2) evident reduction in fluid production; and 3) presence of granulation tissue. At this point, all wounds were surgically closed regardless of their size; when sternal re-approximation was needed, sternal reconstruction was performed using the technique described by Robicsek et al.^[8]^, employing multiple steel wires and/or nitinol clips. In the presence of marked tissue loss, as in cases of extensive bone resection for osteomyelitis, the sternal wound was closed with the aid of pectoral muscle flaps. Our policy has been not to discharge any patient with the VAC system *in situ*.

### Statistical Analysis

Categorical data were described by absolute and relative frequency, whereas continuous variables were tested for normality of distribution and presented as median. Patients undergoing VAC treatment (n=77) were divided into 2 groups: those with superficial SWD (Group 1) and those with deep SWD (Group 2). The outcome variable was the duration of VAC therapy in both groups and in the entire population. Univariate analyzes were performed by Spearman’s correlation analysis (analyzing age, body mass index [BMI], creatinine, ejection fraction) indicating rho value, and by Mann-Whitney test (exploring gender, smoking, diabetes, chronic obstructive pulmonary disease [COPD], arteriopathy, hypertension, *Staphylococcus aureus* infection, use of nitinol clips, bleeding and neurological complications) indicating median and range. Group 1 patients were further divided into 3 similar subgroups according to duration of VAC: 1 to 7 days (median 6 days; subgroup 1), 8-16 days (median 11.5; subgroup 2) and >17 days (median 20 days, subgroup 3). Finally, subgroups and Group 2 were compared with days after VAC using Kruskall-Wallis test followed by Mann-Whitney test. Differences were considered significant at *P*<0.05. All analyses, descriptive and inferential, were performed using the IBM SPSS v.24 software.

## RESULTS

### Patient Data

Out of the 2,890 patients undergoing open-heart procedures during the study period, we identified a total of 92 patients (3%) who had postoperative SWD and whose demographic and surgical data are summarized in Table 1. Seven patients developing mediastinitis were included in deep SWD. During the same interval, 15 patients (16%) with superficial SWD were treated with direct surgical closure without NPWT; these patients were excluded from comparison because of the small number. There were mostly men (n=59, 64%), with a median age of 71 years (range 41-91 years). Median BMI was 26.4 kg/m^2^ (range 20.5-40). Diabetes, mostly non-insulin dependent, was present in 44% of subjects. The median logistic EuroSCORE 1 was 5.6% (range 0.8-64.2) and median EuroSCORE 2 was 2.2% (range 0.5-23.9).

**Table 1:** Preoperative profile and operative characteristics of patients with sternal wound complications.

Characteristics	Number (%)	Median, range
Age (years)		71 (41-91)
Sex		
Male	59 (64)	
Female	33 (36)	
Hypertension	84 (91)	
BMI (kg/m2)		26.4 (20.5-40.1)
Active endocarditis	1 (1)	
Myocardial infarction	19 (21)	
Diabetes		
Oral therapy	29 (31)	
Insulin	12 (13)	
Extracardiac arteriopathy	26 (28)	
COPD	19 (21)	
Creatinine (mg/dL)		1 (0.4-5.3)
LVEF (%)		55 (25-72)
Risk scores (%)		
EuroSCORE 1		5.6 (0.8-64.2)
EuroSCORE 2		2.2 (0.5-23.9)
Type of surgery		
Coronary	74 (80)	
Root	6 (6)	
Ascending	7 (8)	
Mitral valve	5 (5)	
Aortic valve	16 (17)	
Operative times (min)		
CPB		69 (0-309)
X-clamp		43 (0-233)

BMI=body mass index; COPD=chronic obstructive pulmonary disease; CPB=cardiopulmonary bypass; LVEF=left ventricle ejection fraction

### Postoperative Complications

**Fig. 1:**
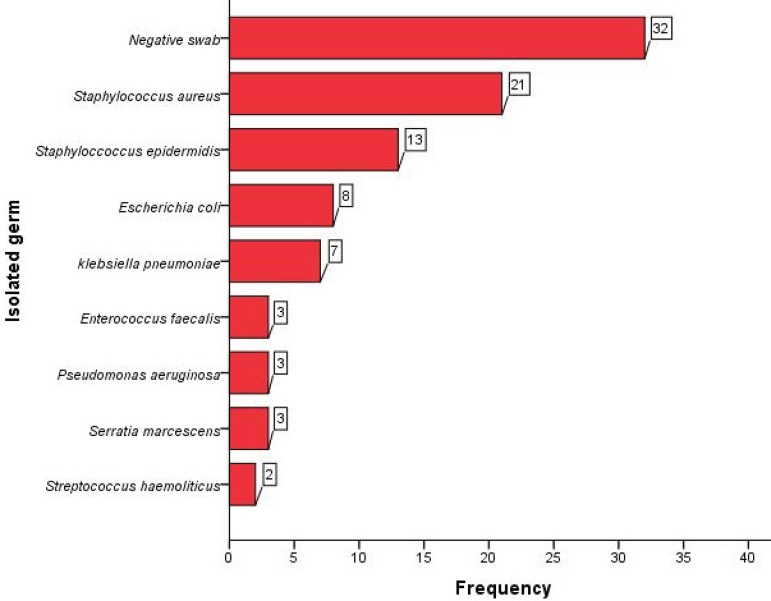
Diagram showing the absolute frequency of negative cultures and isolated germs in the study population.

Table 2 shows the main postoperative complications and the type of treatment. Reoperation for bleeding or cardiac tamponade was needed in 5 patients (5%), dialysis in 6 (7%), while a neurological event (transient ischemic attack in 2 and stroke in 1) occurred in 3 (3%). Superficial and deep SWD (including mediastinitis) occurred in 72 (78%) and 20 (22%) patients, respectively. Figure 1 summarizes the microorganisms involved, showing a prevalence of *staphylococci* and the number of negative cultures.

**Table 2:** Postoperative data, including wound complications and treatment.

Characteristics	Number (%)	Median (range)
Reoperation for bleeding	5 (5)	
Dialysis	6 (7)	
Neurological event	3 (3)	
Type of dehiscence		
Superficial	72 (78)	
Deep (including mediastinitis)	20 (22)	
VAC therapy	77 (84)	
Muscle flap surgery	12 (13)	
Rewiring	15 (16)	
Nitinol clip	7 (8)	
*S. aureus* infection	21 (23)	
Days of hospitalization		25 (6-117)
Days of VAC therapy		12 (3-74)

VAC=vacuum-assisted closure

### Patient Outcome

**Fig. 2:**
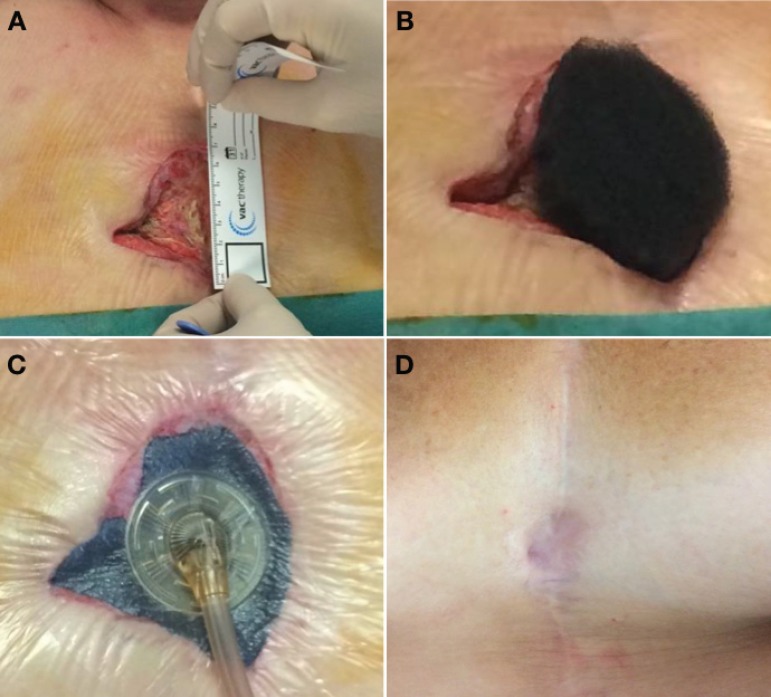
A) Measurement of the wound size; B) Tailoring of the foam; C) Vacuum therapy device in place; D) Final results two years after 19 days of vacuum therapy and direct surgical closure with acceptable cosmetic results.

Median duration of VAC therapy was 12.5 days (from 3 to 74 days); median hospital stay was 25.5 days (from 6 to 117 days). In all cases, complete wound healing was obtained, with good cosmetic results (Figure 2).

Univariate analyzes of risk factors influencing VAC duration are represented in Table 3. In both Group 1 (superficial SWD) and Group 2 (deep SWD), none of the preoperative characteristics examined showed association with longer duration of VAC therapy. In Group 2, there was a trend for postoperative bleeding and neurological complications to be associated with longer treatment duration. In the entire series, staph infection only resulted in a weak predictor of VAC duration.

**Table 3:** Factors influencing days of vacuum-assisted therapy.

	Total (n=77)		Superficial SWD (n=57)		Deep SWD (n=20)	
	**rho or ** **median (range)**	*P* **-value**	**rho or ** **median (range)**	*P* **-value**	**rho or ** **median (range)**	*P* **-value**
Age	0.001	0.997	0.098	0.470	0.227	0.337
BMI	0.070	0.544	–0.026	0.849	0.131	0.581
Creatinine	–0.014	0.904	–0.041	0.763	–0.307	0.188
LVEF	–0.130	0.259	–0.184	0.171	0.157	0.509
Sex		0.824		0.633		0.353
Male	15 (4-45)		10 (3-38)		24 (16-74)	
Female	16 (3-74)		11.5 (4-27)		21 (15-45)	
Smoker		0.671		0.994		0.137
No	17 (4-74)		9.5 (4-38)		25 (17-74)	
Yes	15 (3-45)		12 (3-34)		22 (15-45)	
Diabetes		0.857		0.685		0.288
No	15 (3-56)		12 (3-38)		28 (15-56)	
Yes	16 (4-74)		9.5 (4-27)		23 (16-74)	
COPD		0.601		0.695		0.861
No	15 (3-56)		10 (3-38)		24 (15-56)	
Yes	17 (3-74)		14 (3-23)		24 (17-74)	
Arteriopathy		0.779		0.164		0.939
No	15 (3-45)		12 (3-38)		24 (15-45)	
Yes	17(3-74)		7.5 (3-21)		24 (16-74)	
Hypertension		0.918		0.474		0.433
No	15 (3-19)		15 (13-15)		-	
Yes	16 (3-74)		10 (3-38)		24 (15-74)	
*S. aureus*		0.095		0.383		0.781
No	14 (3-74)		10 (3-27)		24 (16-74)	
Yes	17 (3-45)		15 (3-38)		25 (15-45)	
Nitinol clip		0.414		0.134		0.931
No	15 (3-74)		10 (3-34)		24 (15-74)	
Yes	17 (6-38)		17 (6-38)		-	
Bleeding		0.463		0.484		0.111
No	15 (3-74)		10 (3-34)		24 (15-74)	
Yes	17 (4-45)		15 (4-38)		45 (25-45)	
PNC		0.460		0.896		0.098
No	15 (3-56)		11 (3-38)		24 (15-56)	
Yes	21 (6-74)		13.5 (6-21)		-	

BMI=body mass index; COPD=chronic obstructive pulmonary disease; LVEF=left ventricular ejection fraction; PNC=postoperative neurological complications; SWD=sternal wound dehiscence

**Fig. 3:**
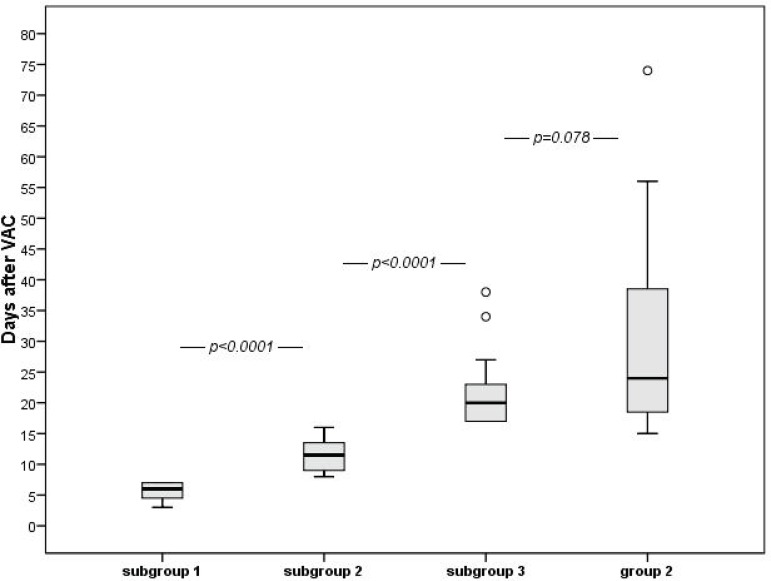
The box plot indicates the length of hospital stay after VAC weaning until discharge, based on days of treatment. Median post-VAC hospital stay was 6 days in subgroup 1, 11.5 days in subgroup 2 and 20 days in subgroup 3 (P<0.0001 between subgroups). Comparison between Group 2 and subgroup 3 of Group 1 showed no statistical significance (P=0.078).

In each subgroup of Group 1 and in Group 2, to better evaluate the treatment efficacy and its influence on length of hospital stay, days of NPWT were compared with duration of hospitalization following VAC insertion until discharge. Median post-VAC hospital stay was 6 days in subgroup 1, 11.5 days in subgroup 2 and 20 days in subgroup 3 (*P*<0.0001 between subgroups). Comparison of post-VAC hospital stay between Group 2 and subgroup 3 of Group1 patients showed no statistical significance (*P*=0.078) (Figure 3).

There were 5 hospital deaths, 2 from myocardial infarction, 2 from stroke and 1 from sepsis, with an overall mortality of 5%. Median follow-up of 87 patients discharged was 3 years (1 to 11 years) and 98% complete. There were 4 late deaths, none directly related to wound complications: 2 patients died of heart failure after 6 and 9 years from their first intervention, 1 patient from pulmonary neoplasm, and 1 from fatal stroke. No cases of SWD recurrence were observed.

### Comment

Sternal wound complications in patients undergoing cardiac surgery, ranging from superficial dehiscence to mediastinitis, have been clearly associated with a higher mortality rate, longer hospital stay and increased risk of other infections, such as those associated with prolonged hospitalization and catheter-related bacteremia^[9-12]^. Moreover, wound infections have been shown to have an important impact on surgical costs related to length of hospital stay, repeated procedures and possible lifelong financial and psychosocial sequelae^[13,14]^.

Several risk factors for SWD have been recognized, the most frequent being obesity, diabetes and a long preoperative hospitalization, some of which may not be easily modifiable^[13,15]^. Most likely for such reason, in the past years the incidence of SWD has remained largely stable, ranging from 2% to 10% in most series^[10,11]^: these data were confirmed in the present study, where we have observed an overall incidence of 3% of SWD. Recently, however, a significantly lower incidence of SWD (1%) has been reported^[1]^, probably because of the introduction of tight glycemic control^[16]^ and other prevention measures indicated by an expert consensus on SWD treatment^[17]^. However, in such multicenter study, the postoperative period considered was limited to 65 days and thus additional events might have been missed or underestimated^[1]^.

So far, the treatment of SWD has been based on different techniques, such as surgical revision with open dressings or closed irrigation, followed by simple closure or more complex procedures as sternal refixation associated with the use of omental or pectoral muscle flaps^[2]^. In 1997, the initial clinical experience with VAC as a new method for wound treatment was reported^[3]^; subsequently, NPWT was also applied to sternal wounds with favorable results^[18,19]^. Moreover, several recent studies comparing VAC with standard treatments have demonstrated the superiority of VAC, concluding that VAC treatment should be considered a bridge to surgical sternal closure^[20,21]^ or as definitive therapy in patients with SWD^[22]^.

In this report, we sought to evaluate the effectiveness of the routinely applied NPWT in a series of consecutive patients undergoing various open-heart procedures through a standard median sternotomy. Our results demonstrate the effectiveness of NPWT, as shown by wound healing and absence of recurrences in all cases. In our analysis, we failed to identify any specific preoperative characteristic influencing VAC duration regardless of SWD type; however, staph infection appears to affect duration of treatment in the entire group, although it has not reached statistical significance. *S. aureus* was the most commonly isolated germ from wound cultures in this study, although still less frequently than reported by others^[15,23,24]^.

As expected, the duration of treatment affected the length of hospitalization. When NPWT lasted more than 8 days (subgroups 2 and 3 of Group 1), post-VAC hospital stay was significantly longer than in subgroup 1. In Group 2 patients, there was a trend for a longer postoperative period compared to subgroup 3 of Group 1; between such patients, however, the difference was not statistically significant, indicating that a deep SWD behaves like a more complex superficial one. We believe that early use of NPWT in the onset of early signs of wound complications and the strict NPWT management and suspension protocol we have followed contributed to the excellent results. However, a longer duration of VAC treatment, besides obviously reflecting the severity of SWD, could also favor the onset of other complications and morbidities, even if unrelated to SWD, which may have an impact on longer hospital stay. Recent data suggest that negative microbiological results are not mandatory to stop VAC treatment in the presence of a clean, granulating wound^[25]^. Others believe that prolonged unnecessarily use of NPWT might have a damaging effect on tissues, thus explaining recurrences of SWD^[15,22]^, which, however, we have not observed. Therefore, after this initial experience, we are considering the possibility to revise our treatment protocol for SWD to render it less strict and simpler but equally effective.

This is a retrospective study, which may be considered one of its main limitations. An additional limitation may be the absence of a contemporary control group treated with more traditional methods, since after introduction of the NPWT in our institution, most patients were managed with this method. Indeed, during the same time interval, a small group of patients with superficial SWD was treated with immediate direct surgical closure, without the use of VAC; these patients were also excluded from the comparison because of the limited number. However, this is a single center experience, which ensures uniformity in diagnosis, patient treatment and accuracy of results, providing adequate information on the use and effectiveness of VAC therapy in patients with SWD.

All patients included in the present study were managed prior to the publication of the recent expert consensus on prevention and management of sternal wound infections. According to these guidelines^[17]^, we have currently adopted new treatment methods such as avoidance of bone wax on the cut edges of the sternum, where topical antibiotics are applied, and a stricter glycemic control.

## CONCLUSION

Our experience has shown that patients with SWD benefit from early and aggressive treatment. This has been witnessed by optimal results in all cases, with complete wound healing and absence of SWD recurrence at follow-up. The use of NPWT has significantly improved patient outcome and represents a fundamental tool in treating subjects with both superficial and deep SWD. The possibility of changing the treatment protocol currently employed at our institution to a less aggressive one, including earlier suspension of NPWT, must be confirmed by further studies.

**Table:** 

Authors' roles & responsibilities	
ADM	Substantial contributions to the conception or design of the work; or the acquisition, analysis, or interpretation of data for the work; drafting the work or revising it critically for important intellectual content; final approval of the version to be published
FDR	Acquisition, analysis, or interpretation of data for the work; final approval of the version to be published
GF	Acquisition, analysis, or interpretation of data for the work; final approval of the version to be published
RM	Acquisition, analysis, or interpretation of data for the work; final approval of the version to be published
GR	Agreement to be accountable for all aspects of the work in ensuring that questions related to the accuracy or integrity of any part of the work are appropriately investigated and resolved; final approval of the version to be published
UB	Substantial contributions to the conception or design of the work; or the acquisition, analysis, or interpretation of data for the work; drafting the work or revising it critically for important intellectual content; final approval of the version to be published
